# Effect of low-dose spironolactone on resistant hypertension in type 2 diabetes mellitus: a randomized controlled trial in a sub-Saharan African population

**DOI:** 10.1186/s13104-016-1987-5

**Published:** 2016-03-23

**Authors:** Romance Nguetse Djoumessi, Jean Jacques N. Noubiap, Francois Folefack Kaze, Mickael Essouma, Alain Patrick Menanga, Andre Pascal Kengne, Jean Claude Mbanya, Eugene Sobngwi

**Affiliations:** Department of Internal Medicine and Specialties, Faculty of Medicine and Biomedical Sciences, University of Yaoundé I, Yaoundé, Cameroon; Department of Medicine, Groote Schuur Hospital and University of Cape Town, Cape Town, South Africa; Medical Diagnostic Center, Yaoundé, Cameroon; Division of Medicine, Sangmelima’s Reference Hospital, Sangmelima, Cameroon; Non-Communicable Diseases Research Unit, South African Medical Research Council and University of Cape Town, Cape Town, 7505 South Africa; Laboratory for Molecular Medicine and Metabolism, Biotechnology Center, University of Yaoundé I, Yaoundé, Cameroon; Yaoundé Central Hospital and Faculty of Medicine and Biomedical Sciences, National Obesity Center, University of Yaoundé I, Yaoundé, Cameroon

**Keywords:** Spironolactone, Resistant hypertension, Type 2 diabetes mellitus, Sub-Saharan Africa, Cameroon

## Abstract

**Background:**

Low-dose spironolactone has been proven to be effective for resistant hypertension in the general population, but this has yet to be confirmed in type 2 diabetic (T2DM) patients. We assessed the efficacy of a low-dose spironolactone on resistant hypertension in a sub-Saharan African population of T2DM patients from Cameroon.

**Methods:**

This was a four-week single blinded randomized controlled trial in 17 subjects presenting with resistant hypertension in specialized diabetes care units in Cameroon. They were randomly assigned to treatment with a daily 25 mg of spironolactone (n = 9) or to an alternative antihypertensive regimen (n = 8), on top of any ongoing regimen and prevailing lifestyle prescriptions. They were seen at the start of the treatment, then 2 and 4 weeks later. The primary outcome was change in office and self-measured blood pressure (BP) during follow-up, and secondary outcomes were changes in serum potassium, sodium, and creatinine levels.

**Results:**

Compared with alternative treatment, low-dose spironolactone was associated with significant decrease in office systolic BP (−33 vs. −14 mmHg; *p* = 0.024), and in diastolic BP (−14 vs. −5 mmHg; *p* = 0.006). After 1 month of spironolactone, all the patients were controlled based on BP below 130/80 mmHg, with significant office BP reduction from 158 ± 17/86 ± 11 to 125 ± 11/72 ± 8, vs. 158 ± 8/94 ± 8 to 144 ± 17/89 ± 12 mmHg in the alternative treatment group. There was no significant variation in sodium and creatinine levels in both groups, but a mild increase of potassium levels in the spironolactone group.

**Interpretation:**

Add-on low-dose spironolactone was effective in reducing BP to optimal levels in T2DM Cameroonian patients despite mild increase in serum potassium.

*Trial registration* ClinicalTrials.gov Identifier *NCT02426099*. *Date of registration* April 2015

## Background

Hypertension and diabetes have reached epidemic proportions worldwide, fuelling a burden of cardiovascular disease. It is estimated that at least 1 billion adults have hypertension globally, a figure that is projected to increase to 1.5 billion by 2025 [1]. The International Diabetes Federation (IDF) has estimated that the number of adults with diabetes in the world will increase by 55 %, from 381.8 million in 2013 to 591.9 million in 2035 [2]. Hypertension is very frequent in diabetes patients. It affects up to 70 % of individuals with diabetes and is approximately twice more common in individuals with diabetes than in those without [3]. Coexistence of diabetes and hypertension is associated with increased cardiovascular morbidity and mortality [4–6].

Blood pressure (BP) control is a huge challenge in diabetic patients, especially those of African ancestry. To date, there is a general consensus on angiotensin converting enzyme (ACE) inhibitors as first-line treatment for hypertension in diabetic subjects. Diuretics, B blockers and calcium channel blockers (CCB) can be added as second or third-line treatment to reach the target BP objectives. The choice of these drugs generally takes account of existing cofactors or indications [7]. More than 50 % of diabetic patients with hypertension do not reach target BP control levels, sometimes in spite of double, triple, or even quadruple drug therapies [7]. This is partly due to resistant hypertension, defined as a failure of appropriate treatment with antihypertensive drugs from three or more classes, one of which a diuretic, to lower BP to the target level [8].

The prevalence of resistant hypertension is estimated at 10–15 % in all patients treated with antihypertensive depending on definition and study population [9–12]. It is particularly prevalent in diabetes patients. The cause of resistant hypertension is in most cases multifactorial, including factors such as poor adherence, obesity, vascular stiffening, chronic renal disease as well as an underlying endocrine disease like primary aldosteronism; a condition present in 14 % of type 2 diabetes mellitus (T2DM) patients with resistant hypertension [13]. In addition to its classic effects on sodium-water retention and excretion of potassium and magnesium, it has been proven that aldosterone has cardiovascular effects like myocardial fibrosis, cardiac arrhythmia and endothelial dysfunction [14–16]. During the last decade, studies like RALES (spironolactone) and EPHESUS (eplerenone) have provided reliable evidences on the efficacy of low doses of aldosterone antagonists in reducing the morbidity and mortality of severe heart failure and the control of resistant hypertension with low doses of spironolactone as add-on therapy [17, 18]. However, there is still scarce data on the benefit of spironolactone in diabetic patients, especially those of African ancestry. To address this clinical issue, we conducted a pilot prospective randomized trial to evaluate the effect of low dose spironolactone on BP control in a group of T2DM Cameroonian patients with resistant hypertension.

## Methods

### Ethical considerations

The study protocol was approved by the National Research Ethics Committee for Human Health of Cameroon (Ethical approval N° 077/CNE/SE/2012). Written informed consent was obtained from all participants. The study was conducted in accordance with the Helsinki Declaration.

### Protocol and registration

This project was retrospectively registered on ClinicalTrials.gov in April 2015. *NCT02426099*. A protocol was developed during the planning process.

### Design

It was a prospective randomized controlled single blinded trial conducted over a six-month period from October 2011 to March 2012. There was no change in protocol during the study.

### Centers and patients

The reference population comprised diabetic patients aged <75 years presenting with a resistant hypertension of unknown etiology at the outpatient clinics of the National Obesity Center and of the Diabetes Care and Education Center of the Yaoundé Central Hospital, as well as those participating in an ongoing study on glycated hemoglobin (HbA1c). Resistant hypertension was defined as an office BP value ≥140/90 mmHg and self-blood pressure measurement (SBPM) ≥130/80 mmHg under at least three antihypertensive drugs at optimal dosages for at least two months, including a diuretic [8]. From this population, those who consented were further excluded if they fulfilled any of the following criteria: T2DM with overt acute/chronic complications, serum potassium ≥5.5 mmol/l, estimated Glomerular Filtration Rate (eGFR) calculated using the Modification of Diet in Renal Disease formula ≤30 ml/min/1.73 m^2^ of body weight, absolute contraindication to any of the drug regimen of the trial, and current aldosterone antagonist treatment or cessation within the last 15 months.

### Randomization and blinding

The experimental population was divided into two groups according to a restricted randomization method of blocks [19]. It consisted of drawing without replacement one out of two types of non-distinguishable counters from a non-transparent bag. Depending on the type of counter drawn, the subject was assigned to the spironolactone group or to the control group (taking an alternative drug regimen) by researchers who were aware of the type of counter presented. The alternative drug regimen included: candesartan, atenolol and alpha methyldopa; and the choice to administer each depended on the respective absolute/relative contraindications [20] applicable to the subject. As from the randomization day, subjects allocated to the spironolactone group received a daily 25 mg tablet of spironolactone, whereas those from the control group received either a daily 100 mg of atenolol, a daily 8 mg of candesartan, or a daily 750 mg of alpha methyldopa, all of these drugs added to their previous regimen, with unchanged diet. They were followed up during visits scheduled at the second and fourth weeks of treatment.

### Outcomes measures

A clinical examination was done and laboratory measurements carried out before the intervention (baseline) and at follow-up visits. Clinical examination included BP measurement (at baseline and during all follow-up visits), an electrocardiogram (ECG) and anthropometric parameters recorded at baseline. Biochemical measures included: serum sodium, serum potassium, serum creatinine, fasting capillary glycaemia, the lipid profile [including blood cholesterol, triglyceride, high density lipoprotein cholesterol (HDL), and low density lipoprotein cholesterol (LDL)], blood urea nitrogen (BUN) and proteinuria. All biochemical measurements were done at baseline and serum sodium, potassium and creatinine levels were checked again during visits.

Office BP and self-blood pressure measurement (SBPM) were considered at every visit. For office BP, three serial measurements taken 5 min apart in the sitting position were obtained from the left arm placed at the level of the heart, using an automated sphygmomanometer Omron HEM-705 CP (Omron Corporation, Tokyo, Japan). The average of the second and third measurements was used for all analyses. The daytime SBPM considered was the mean of BP values self-recorded at home in the same condition than those applied in clinic three consecutive days prior to the visit, without health personnel. ECG was recorded using the CardiMax FX-7302 electrocardiograph (Fukuda DenShi, Tokyo, Japan). Weight was measured in light clothed subjects to the nearest 0.5 kg with a mechanical scale; height was measured in the upright position to the nearest 0.5 cm, and body mass index (BMI in kg/m^2^) was calculated as $weight (cm)/[height \left( m\right)x \,height\left( m \right)]$. Waist circumference was measured at the horizontal plane midway between the lower rib margin and the iliac crest with a measuring tape. Serum sodium, serum potassium, serum creatinine, BUN, serum triglyceride, serum cholesterol and serum HDL cholesterol were measured using standard colorimetric procedures. LDL cholesterol was calculated with the Friedwald’s formula [21]. Proteinuria was obtained by dipstix of spot urine, and considered positive for at least 1+. Fasting glycaemia was recorded using the Accu-Chek^®^ Compact Plus glucometer (F. Hoffmann-La Roche AG, Basel, Switzerland). Unfortunately aldosterone was not measured in this study.

All drug information were checked at each visit by interviewing the participants and they were invited to phone the research team if any change was noticed in-between visits.

### Statistical analysis

We needed at least eight individuals in each group in order to detect with 80 % power (ϕ) and 5 % risk of error (α) a minimal relevant difference (δ) of 20 (SD 4) mmHg [22] for the systolic blood pressure (SBP) between the two groups, considering no dropout, based on the following formula [23]:

$$n = \left[ {\frac{{\sigma \left( {\rm{Z}_{{1\frac{\alpha }{2}}} + \rm{Z}_{\upphi } } \right)}}{\delta }} \right]^{2}$$

Data were analyzed based on the intention to treat principle, using IBM SPSS for Windows, version 20.0 (IBM Corp., Armonk New York, USA). Continuous variables are expressed as means with standard deviation (SD), and categorical variables as count (percentage). Non parametric Mann–Whitney tests were used to compare continuous variables. A *p* value <0.05 was considered statistically significant.

## Results

**Fig. 1:**
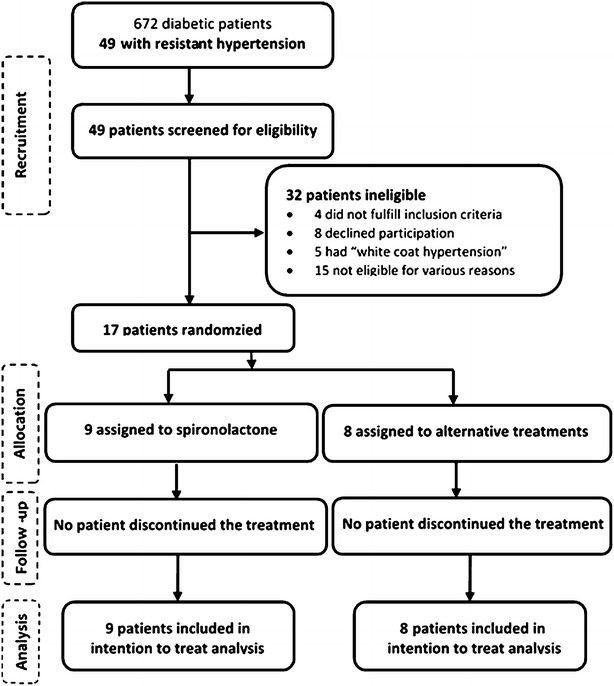
Trial profile

Out of the 672 diabetic patients received at the enrolment centers between October 2011 and March 2012, 377 (56.1 %) were hypertensive; of whom 49 (13 %) had a diagnosis of resistant hypertension were screened for eligibility. After exclusion of ineligible subjects, 17 patients were definitively randomized. None of the participants left the trial, and all of them were included in the intention-to-treat analysis. There was a good compliance to treatment and no change in medications occurred during the follow up. The trial profile is outlined in Fig. 1.

As expected, the profile of participants was similar across the two trial arms at baseline. There were no sex differences between the two groups, although this has to be interpreted in the context of small numbers. The mean office SBP was 158 mmHg in both groups. Mean serum potassium, sodium and creatinine levels were within normal ranges in both groups. All trial participants had chronic kidney disease (CKD) according to the KDIGO definition as the persistence for at least 3 months of an estimated glomerular filtration rate below 60 ml/min/1.73 m^2^ [24]. Other characteristics are summarized in Table 1. Before the intervention, all trial participants were on treatment regimens comprising an ACE inhibitor, a diuretic and a calcium channel blocker. Besides this triple therapy, five subjects had a fourth drug, which was either alpha or beta-blockers; and the mean daily dosage was appropriate in both groups for each drug (data not shown).

**Table 1:** Baseline characteristics of the study population

	Spironolactone (n = 9)	Alternative drug regimen (n = 8)	*p* value
Sociodemographic and clinical characteristics			
Age (years)	64.6 (9.6)	61.0 (6.6)	0.15
Sex (male/female)	3/6	5/3	0.34
Smoking	0	0	–
Alcoholic index (g/l)	0	0	–
Body mass index (kg/m^2^)	30.3 (5.4)	30.5 (7.7)	0.95
Waist circumference (cm)	104.8 (12.5)	102.7 (10.2)	0.74
Office SBP^a^ (mmHg)	158 (17)	158 (8)	0.96
Office DBP^a^ (mmHg)	86 (11)	94 (8)	0.08
Office heart rate (beats/minute)	75 (12)	76 (1) 6	0.92
Self-measured SBP (mmHg)	140 (9)	151 (15)	0.06
Self-measured DBP (mmHg)	78 (8)	91 (15)	0.07
Biological parameters			
Fasting glycaemia (g/l)	182.89 (105.45)	141.13 (56.36)	0.33
Glycatedhaemoglobin (%)	8.45 (1.76)	8.64 (2.43)	0.88
Blood urea nitrogen (g/l)	0.48 (0.01)	0.48 (0.04)	0.96
Serum creatinine (mg/l)	15.63 (5.97)	12.45 (3.41)	0.20
eGFR (ml/min/1.73 m^2^)	55.25 (23.76)	73.99 (27.96)	0.15
Serum potassium (mmol/l)	4.02 (0.15)	4.01 (0.10)	0.88
Serum sodium (mmol/l)	140.5 (0.5)	141.0(0.7)	0.11
Serum total cholesterol (g/l)	1.71 (0.39)	2.21 (1.00)	0.43
Serum HDL-C (g/l)	0.58 (0.09)	0.35 (0.10)	0.06
Serum LDL-C (g/l)	0.88 (0.35)	1.54 (1.20)	0.43
Serum triglyceride (g/l)	1.32 (0.98)	1.63 (1.43)	0.90
Proteinuria (number of +)	1.11 (1.6)	1 (1.06)	0.84
Complications			
Chronic kidney disease	9 (100)	8 (100)	

Data are expressed as mean (standard deviations); no statistical difference was found for any of the parameters compared between the spironolactone and control groups; Alternative drug regimens include atenolol, candesartan and alpha methyldopa

Current smoking or ceased within the last 3 years

Chronic kidney disease which is the only categorical variable is expressed in number (percentage)

*SBP* systolic blood pressure; *DBP* diastolic blood pressure

*eGFR* estimated glomerular filtration rate using the modification of diet in renal disease equation; *HDL-c* high density lipoprotein cholesterol; *LDL-c* low density lipoprotein cholesterol

^a^ Expressed as the average of the second and third measurements

The mean change in office and self-measured systolic and diastolic BP at the end of the trial is presented in Table 2. The target BP was defined in this study by systolic (and diastolic) BP <130 mmHg (and 80 mmHg) as recommended by the IDF up to 2012 [25]. Within the spironolactone group, there were significant reductions in systolic and diastolic office BP and in systolic SBPM. Their mean systolic and diastolic office BP decreased respectively from 158 ± 17 mmHg to 125 ± 11 mmHg (*p* = 0.009) and from 86 ± 11 to 72 ± 8 mmHg (*p* = 0.009). Only one out of nine participants in the spironolactone group did not reach the target office BP (with BP = 140/67 mmHg 1 month after); defined by systolic (and diastolic) BP <140 (and 90 mmHg). However, the target for SBPM, defined by systolic BP (and diastolic) <135 (and 85 mmHg) was reached after 1 month for this patient, as all the participants in the spironolactone group. Comparing the two groups, the systolic and diastolic BP, both office-based and self-measured, decreased more in the spironolactone group than in the control group, with the systolic and diastolic office BP respectively decreasing by 33 vs. 14 mmHg (*p* = 0.024) and by 14 vs. 5 mmHg (*p* = 0.006). In the control group, the target BP was achieved neither for office BP [end-of-trial mean BP (mmHg) = 144 ± 17/89 ± 12], nor for SBPM [end-of-trial mean BP (mmHg) = 142 ± 14/86 ± 14]. Only two out of eight participants in the control group reached the target control BP levels.

**Table 2:** Comparison of blood pressure change after intervention among the two groups and within each group

	Spironolactone (n = 9)	Alternative drug regimen (n = 8)	*P* value
Office blood pressure			
SBP(mmHg)			
Before	158 (17)	158 (8)	0.96
After	125 (11)	144 (17)	0.02
*p* value	0.01	0.08	
DBP(mmHg)			
Before	86 (11)	94 (8)	0.08
After	72 (8)	89 (12)	0.01
*p* value	0.01	0.02	
Self-blood pressure measurement			
SBP(mmHg)			
Before	140 (9)	151 (15)	0.06
After	123 (10)	142 (14)	0.01
*p* value	0.02	0.02	
DBP(mmHg)			
Before	78 (8)	91 (15)	0.07
After	73 (7)	86 (14)	0.04
*p* value	0.11	0.09	

Data are expressed as means (standard deviations)

*SBP* systolic blood pressure; *DBP* diastolic blood pressure

**Fig. 2:**
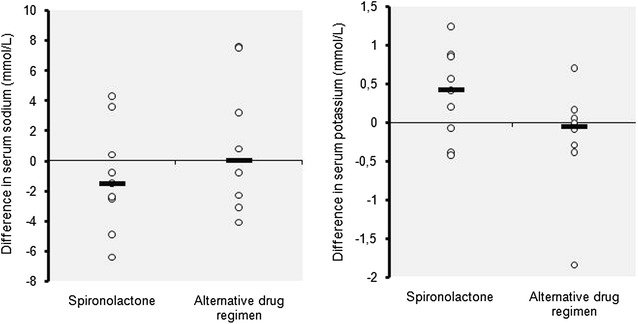
Distribution of individual variation of serum sodium and potassium in each group after intervention

**Fig. 3:**
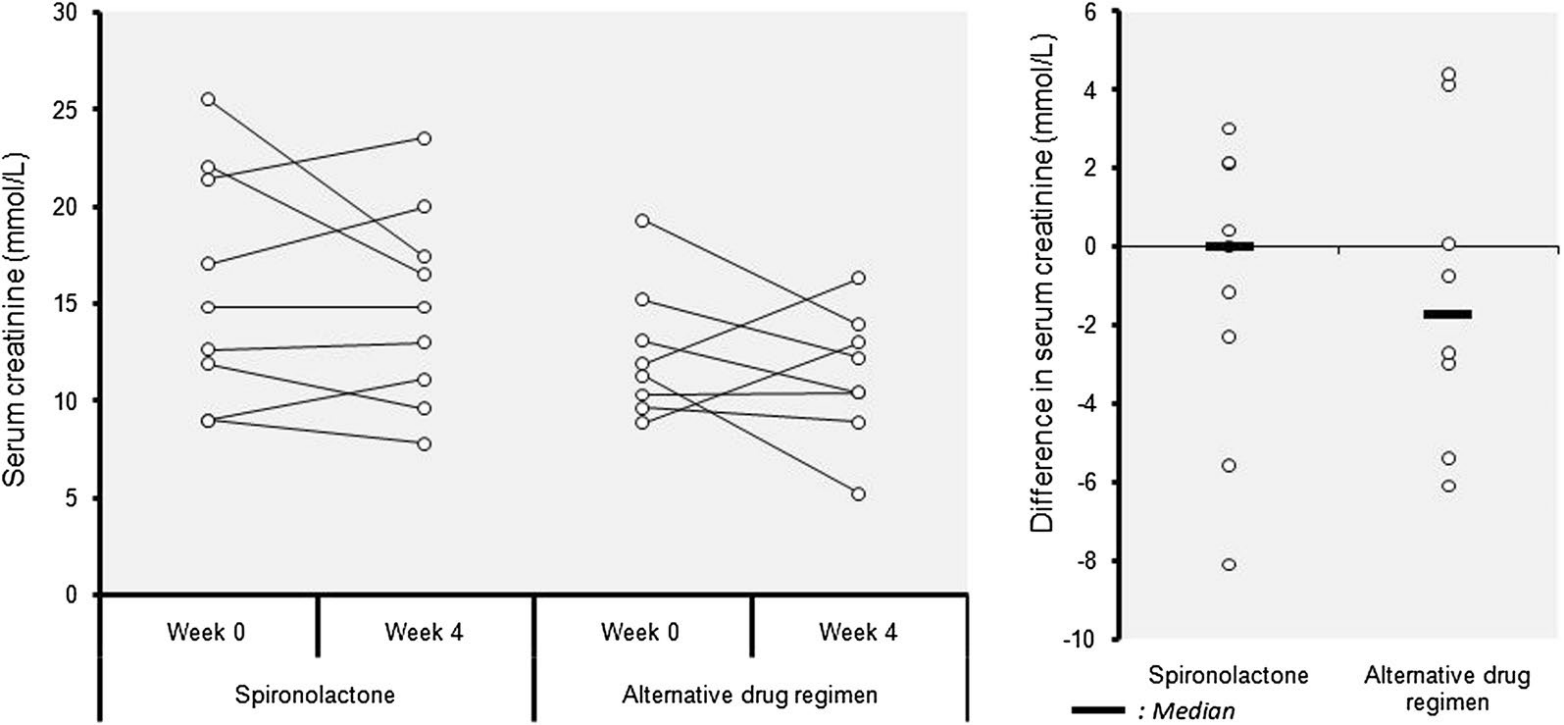
Individual variation of serum creatinine after intervention

The mean serum potassium was mildly increased from 4.02 to 4.38 mmol/L in the spironolactone group after 4 weeks (Fig. 2), the highest value being at 6.28 mmol/l at the end of the trial. But the difference between the two study groups was not significant; an observation made also for serum sodium and creatinine levels (Fig. 3). Adverse drug reactions were observed only in the spironolactone group at the end of the trial. These included: somnolence and asthenia in one patient (11.1 %), and hyperkalemia (at 5.61 and 6.28 mmol/l) in two other patients (22.2 %) without clinical manifestations. However, these two patients had high initial potassium levels of 5.04 mmol/L each. No unexpected adverse drug effect was noted.

## Discussion

Hypertensive patients with diabetes are at increased risk of developing diabetes-specific complications [26]. However, achieving and maintaining optimal BP control in this population remains a challenge [25, 26]. Unlike in the general population where low-dose spironolactone has been shown to be effective for the treatment of resistant hypertension [27–29], little is known on its efficacy in people with diabetes in whom resistant hypertension tends to be highly prevalent and difficult to control. This evidence is much needed for patients of African ethnicity who have been found to be less responsive to the guidelines recommended first line antihypertensive drugs [ACEI or Angiotensin II Receptor Blocker (ARB)] in people with diabetes [25]. Thus, we conducted a randomized controlled trial to evaluate the effect of a low-dose spironolactone as add-on therapy on office and self-measured BP in a group of sub-Saharan African T2DM patients with resistant hypertension from Cameroon. After 1 month of treatment, a daily 25 mg of spironolactone was associated with a significant reduction of both office and SBPM, and high likelihood of reaching recommended target blood pressure, without significant change in serum sodium concentrations, and only a mild elevation of serum potassium levels.

Compared to the alternative drug regimen group, the spironolactone group exhibited a significant reduction of BP. The achieved reduction in their mean office BP (33/14 mmHg) was higher than the 12/7 mmHg reported by *Hase* et al. after treating 25 T2DM Japanese patients for 24 weeks with similar dose of spironolactone [30]. The more pronounced renal impairment in their population could account at least in part to this difference. Differing durations of follow-up is also another possible reason, which would suggest that perhaps, following our cohort beyond 4 weeks could reveal some attenuation of the effect of spironolactone with time. It is of note however that, in Hase et al’s study [30], BP reduction was still significant 2 years after the introduction of spironolactone. *Oxlund* et al. also reported significant reduction in office and ambulatory BP with a fixed daily dose of 25 mg of spironolactone in 57 T2DM Danish patients after 16 weeks of therapy [31]. These results, together with ours, suggest the possible effectiveness of small-dose spironolactone in people with diabetes, unlike the general population or non-diabetic CKD patients in who higher doses are often needed to overcome resistant hypertension [29, 32].

Unlike participants in our alternative drug regimen group, all those in the spironolactone group reached the target office BP of 130/80 mmHg recommended by the IDF [25], with the exception one patient who reached the target diastolic, but not systolic BP. In the study conducted by *Oxlund* et al. [31], only 36 % of patients receiving spironolactone reached the target BP. Although there is continuing debate about the target value of BP in T2DM patients, with some claims that the goal of 130/80 mmHg is less realistic in older populations [26] like ours (mean age 64 years), this target is associated with a reduced overall and cardiovascular mortality in T2DM patients [25], and still seems achievable.

Recent studies in animals and humans, have reported that spironolactone produces many other metabolic benefits (preventing dyslipidemia) and cardiovascular effects in T2DM, beside its known diuretic properties [15, 16, 29]. It reduces vascular stiffness and mediates vasodilation by increasing nitric oxide bioavailability and inhibiting the sympathetic system [33]. These last effects are likely more pronounced in resistant hypertension, where they translate into reduced pulse-wave velocity and systolic BP [29, 34]. This can possibly explain the more pronounced action on systolic BP observed in our study. Alongside the above effects, spironolactone has many other beneficial effects in diabetic patients. In particular, it improves endothelial function, exerts antithrombogenic effects by blocking aldosterone, thereby mediating antithrombotic effects [34]. Furthermore, it reduces the risk of inflammatory cerebral, myocardial, and renal injury; and is associated with a reduced prevalence of arrhythmia, heart failure, sudden death and post myocardial infarction mortality [34]. It has been proven to reduce microalbuminuria [30]. Considering all these actions, spironolactone should perhaps not be conceived anymore as a simple antihypertensive drug, but also as a cardioprotective and nephroprotective drug suitable for diabetic patients, in the absence of other contraindications. Spironolactone probably has a place among the first-line antihypertensive drugs in diabetic patients regardless of whether they have resistant hypertension or not. Indeed, the “aldosterone escape” phenomenon characterized by an initial partially reversible decrease of aldosterone is well known with the use of ACEI agents, and makes aldosterone specific blockers such as spironolactone more effective [34]. *Makhlough* et al. also proved that a low dose spironolactone used alone was as effective as spironolactone-losartan combination in improving on the diabetic nephropathy [35]. These arguments corroborate the fact that it is not an absolute necessity to combine spironolactone which mediates (with better results) all desired effects of ACEI/ARB with any of those drugs. Such associations are not even recommended [25], since they increase the risk of adverse drug reactions, including hyperkalemia [27, 29–32, 35].

Despite the safe dosage of spironolactone used in this study, we observed a mild increase in mean serum potassium levels in the spironolactone group. Even though severe hyperkalemia was noted in two subjects at the end of the study, this was likely driven by their high initial level of serum potassium level. Of note, all those patients had CKD, which is associated with high risk of hyperkalemia [32]. Furthermore, they were all on ACEI which can also increase serum potassium levels. The above suggest that caution should be exercised when prescribing spironolactone to diabetes patients with serum potassium in the upper end of the normal range, or who have concomitant conditions or are receiving treatments that can adversely increase potassium levels. Besides hyperkalemia, we did observe somnolence and asthenia in one patient (11.1 %) in the spironolactone group at the end of the trial. These are known mild effects of the drug [36].

Our study is mostly limited by the small sample size, limiting our ability to generate stable estimates or to perform post hoc subgroups analyses. More adequately powered trials with extended follow-up are needed to draw definitive conclusions. Indeed, since hypertension is a chronic disease, the benefit of low-dose spironolactone in long term treatment of resistant hypertension still needs to be demonstrated.

## Conclusions

This trial has shown that a low-dose (25 mg) of spironolactone as an add-on therapy for resistant hypertension among Black Africans with T2DM was associated with a significant reduction of the office and self-measured BP to optimal levels after 1 month of treatment, without affecting sodium level, but with mild elevation of serum potassium.

## Abbreviations

T2DM: type 2 diabetes mellitus
IDF: International Diabetes Federation
BP: blood pressure
SBPM: self-blood pressure measurement
PA: primary aldoterolism
HbA1c: glycated hemoglobin
eGFR: estimated glomerular filtration rate
HDL: high density lipoprotein
LDL: low density lipoprotein
BUN: blood urea nitrogen
BMI: body mass index
CKD: chronic kidney disease
ACEI: angiotensin enzyme inhibitor
ARB: angiotensin II receptor blocker
